# Molecular mechanism of the anti-inflammatory effects of *Sophorae Flavescentis Aiton* identified by network pharmacology

**DOI:** 10.1038/s41598-020-80297-y

**Published:** 2021-01-13

**Authors:** Naiqiang Zhu, Jingyi Hou

**Affiliations:** Department of Minimally Invasive Spinal Surgery, the Affiliated Hospital of Chengde Medical College, Chengde, 067000 China

**Keywords:** Medical research, Molecular medicine

## Abstract

Inflammation, a protective response against infection and injury, involves a variety of biological processes. *Sophorae Flavescentis* (Kushen) is a promising Traditional Chinese Medicine (TCM) for treating inflammation, but the pharmacological mechanism of Kushen’s anti-inflammatory effect has not been fully elucidated. The bioactive compounds, predicted targets, and inflammation-related targets of Kushen were obtained from open source databases. The “Component-Target” network and protein–protein interaction (PPI) network were constructed, and hub genes were screened out by topological analysis. Gene ontology (GO) and Kyoto Encyclopedia of Genes and Genomes (KEGG) enrichment analyses were performed on genes in the PPI network. Furthermore, nitric oxide (NO) production analysis, RT-PCR, and western blot were performed to detect the mRNA and protein expression of hub genes in LPS-induced RAW264.7 cells. An immunofluorescence assay found that NF-κB p65 is translocated. A total of 24 bioactive compounds, 465 predicted targets, and 433 inflammation-related targets were identified and used to construct “Component-Targets” and PPI networks. Then, the five hub genes with the highest values-IL-6, IL-1β, VEGFA, TNF-α, and PTGS2 (COX-2)- were screened out. Enrichment analysis results suggested mainly involved in the NF-κB signaling pathway. Moreover, experiments were performed to verify the predicted results. Kushen may mediate inflammation mainly through the IL-6, IL-1β, VEGFA, TNF-α, and PTGS2 (COX-2), and the NF-κB signaling pathways. This finding will provide clinical guidance for further research on the use of Kushen to treat inflammation.

## Introduction

Inflammation can be discovered and diagnosed early, and it is associated with huge risks in a variety of diseases, including arthritis^1^, atherosclerosis^2^, Alzheimer’s diseases^3^, inflammatory bowel syndrome^4^, and injury^5^. Cytokines are involved in both inflammation and anti-inflammatory effects via multifunctional molecules depending on the manner of the inflammatory response^6^. Thus, pro-inflammatory cytokines IL-6, tumor necrosis factor-α (TNF-α), and IL-18 initiate and amplify inflammatory processes, whereas anti-inflammatory cytokines, such as IL-10, inflammatory receptor agonist (IRA), and transforming growth factor-β (TGF-β) negatively regulate these processes^7^. Almost all acute and chronic disease are driven or regulated by inflammation, and inflammatory response are fairly complicated processes that involve the synthesis and release of a large number of inflammatory factors. Currently, the anti-inflammatory drugs approved for clinical use include (a) non-steroidal anti-inflammatory drugs (NSAIDs)^8^, such as aspirin, paracetamol, anti-inflammatory drugs, and ibuprofen, and (b) corticosteroid hormones, such as prednisone and steroidal anti-inflammatory drugs such as adrenocorticosteroids and androgens)^9^. However, the long-term use of anti-inflammatory drugs is associated with many side effects and poor prognosis, resulting in serious the gastrointestinal reactions and causing specific damage to certain parts of the cardiovascular system.

Kushen (*Sophorae Flavescentis*) is a dry stem of the medicinal plant of *Sophora flavescens*, which is used to clear heat and dampness from the body and kill insects^10^. Kushen products are abundant in China, and they are produced in a number of provinces, including the Hebei, Henan, Shandong, Anhui, Hubei, and Xinjiang provinces^11^. Clinical trials have shown that Kushen has low toxicity and side effects. Moreover, Kushen exerts a variety of therapeutic effects, such as swelling reduciton, immunostimulation, and anti-arrhythmic, anti-tumor, and anti-bacterial effects^12^. Kushen can act on many key pathways and links in the anti-inflammatory process; however, the specific mechanisms have not been fully explained.

**Figure 1:**
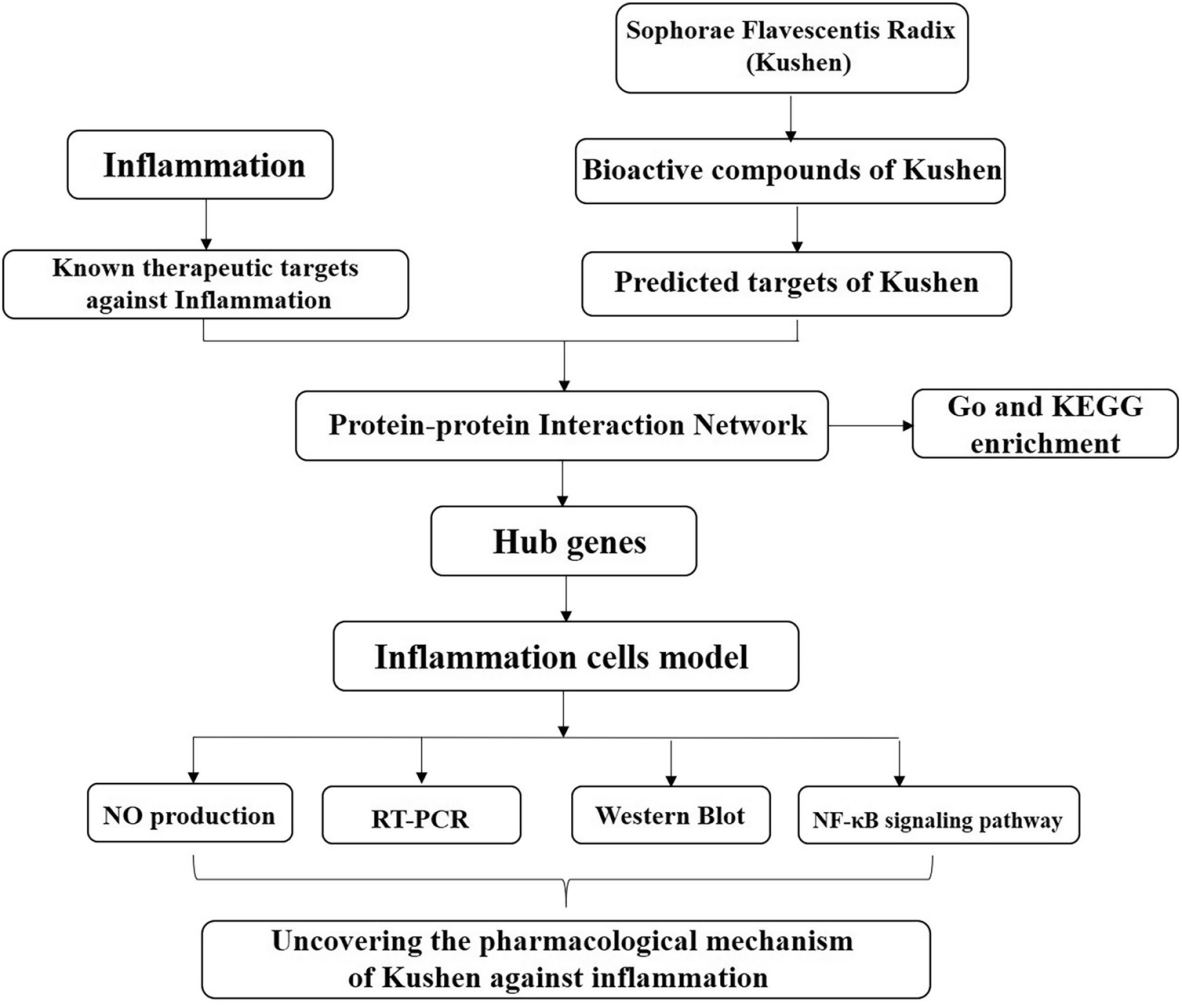
The framework of Kushen extract for the treatment of inflammation.

Network pharmacology is based on the theory of receptors and biological network technology. It analyzes multi-component, multi-target, and multi-pathway synergistic relationships between drugs, targets, and diseases, and then use them to explain drug action^13^. Traditional Chinese Medicine (TCM) operates systematically and holistically, and network pharmacology has allowed TCM research to go beyond focusing on a single ingredient, target, and disease and to study the efficacy of TCM and its mechanisms in depth^14^. In this study, the network pharmacology method was used to predict the effective components and potential targets of Kushen and its hub genes involved in the treatment of inflammation. This network pharmacology approach in combination with molecular biology experiments for further verification provided a basis for future research on the anti-inflammatory mechanism of Kushen (Fig. 1).

## Results

### Identification of bioactive compounds and targets

The TCMSP, ETCM, and SymMap databases were used to determine the bioactive compounds in Kushen. A total of 19 bioactive compounds were identified based on the parameters oral bioavailabilty (OB) ≥ 30% and drug-likeness (DL) ≥ 0.18. Further, several bioactive compounds, whose OB and DL values were below the screening thresholds were excluded. According to the studies, kurarionl, dehydromiltirone, kushenol B, kushenol F, and kushenonl E were also included (Table 1). Next, we found 465 targets corresponding to the bioactive compounds in Kushen (Supplementary Excel 1). Furthermore, OMIM, Genecards, and PubMed-gene were screened using the search terms as anti-inflammatory, inflammation, and anti-inflammation, and 433 targets were found in all three databases (Supplementary Excel 2).

**Table 1:** Information of bioactive compounds of Kushen.

Molecular name	OB (%)	DL	Degree	Structure
Luteolin	36.16	0.25	129	
8-Isopentenyl-Kaempferol	38.04	0.39	13	
Sophoramine	42.16	0.25	11	
Wighteone	42.80	0.36	11	
Hyperforin	44.03	0.6	4	
Quercetin	46.43	0.28	207	
Kushenin	47.62	0.38	49	
Kurarionl	0.95	0.67	5	
Kushenol J_Qt	50.86	0.24	6	
Norartocarpetin	54.93	0.24	3	
Sophoridine	60.07	0.25	9	
Isosophocarpine	61.57	0.25	218	
Matrine	63.77	0.25	18	
Sophocarpine	64.26	0.25	2	
Inermin	65.83	0.54	11	
Cis-Dihydroquercetin	66.44	0.27	4	
Formononetin	69.67	0.21	48	
Dehydromiltirone	24.57	0.26	166	
Inermine	75.18	0.54	11	
Phaseolin	78.20	0.73	16	
Glyceollin	97.27	0.76	12	
Kushenol B	1.21	0.75	7	
Kushenol F	18.85	0.61	7	
Kushenol E	19.66	0.59	7	

### Compound-target network construction and analysis

**Figure 2:**
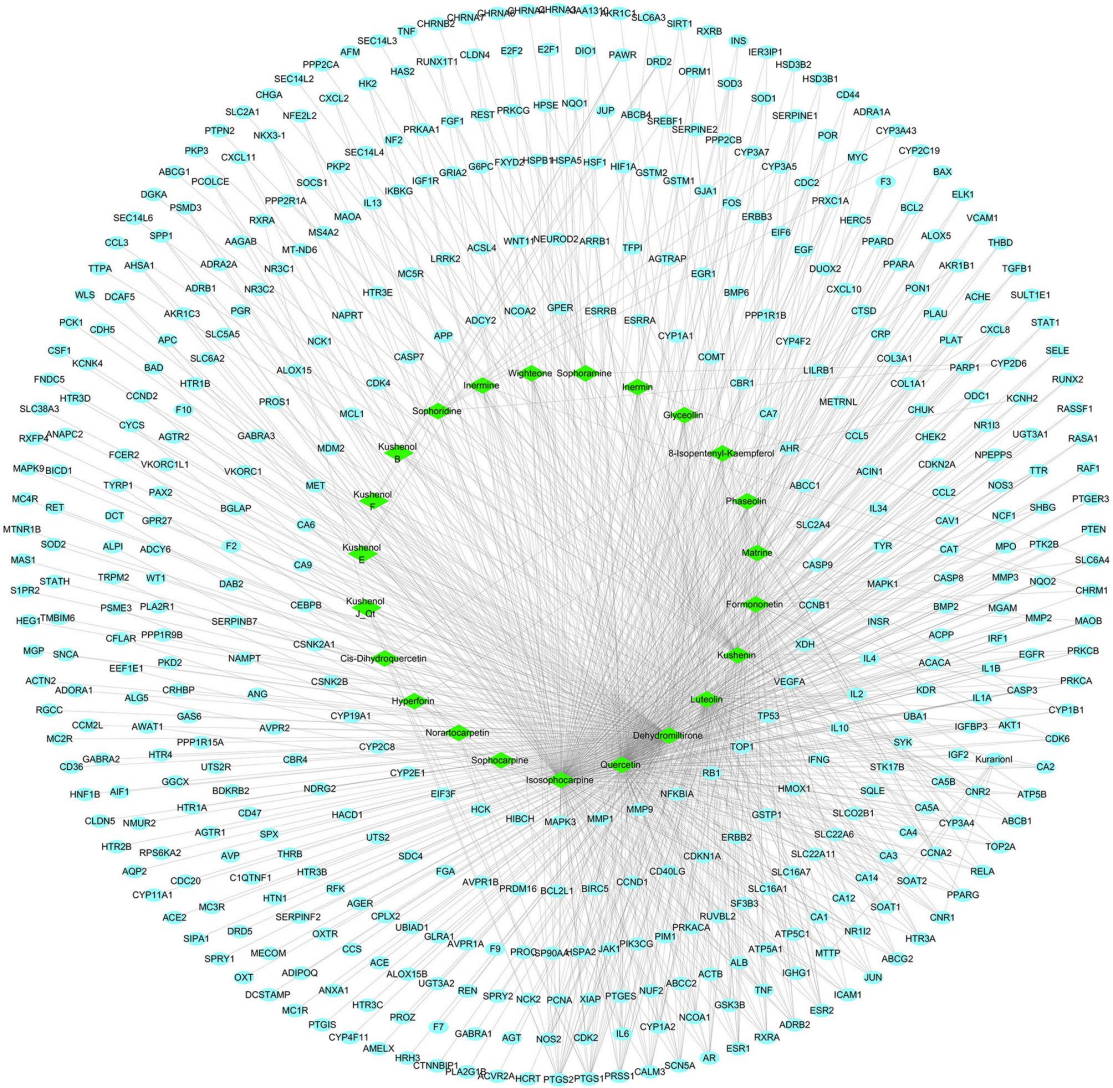
The Compound-Target network of Kushen. Green nodes indicate bioactive compounds, blue nodes indicate target proteins, and edges indicates interaction between ingredients and targets.

As shown in Fig. 2, the compound-target network consists of 489 nodes (24 bioactive ingredients and 465 targets) and 974 interaction edges. In descending order of degree, the top four bioactive ingredients were isosophocarpine (degree 218), quercetin (degree 207), dehydromiltirone (degree 166), and luteolin (degree 129) (Table 2), suggesting these bioactive ingredients are crucial to the pharmacological action of Kushen.

**Table 2:** Hub genes and topological properties.

Gene Symbol	Uniprot ID	Target	Target Class	Degree
IL6	P05231	Interleukin 6	None	70
TNF-α	P01375	Tumor necrosis factor	Signaling	66
VEGFA	P15692	Vascular endothelial growth factor A	Signaling	63
PTGS2	P35354	Prostaglandin-endoperoxide synthase 2	Enzyme	62
IL1β	P01584	Interleukin 1 beta	None	57

### PPI network for Kushen in the treatment of inflammation and hub genes analyses

**Figure 3:**
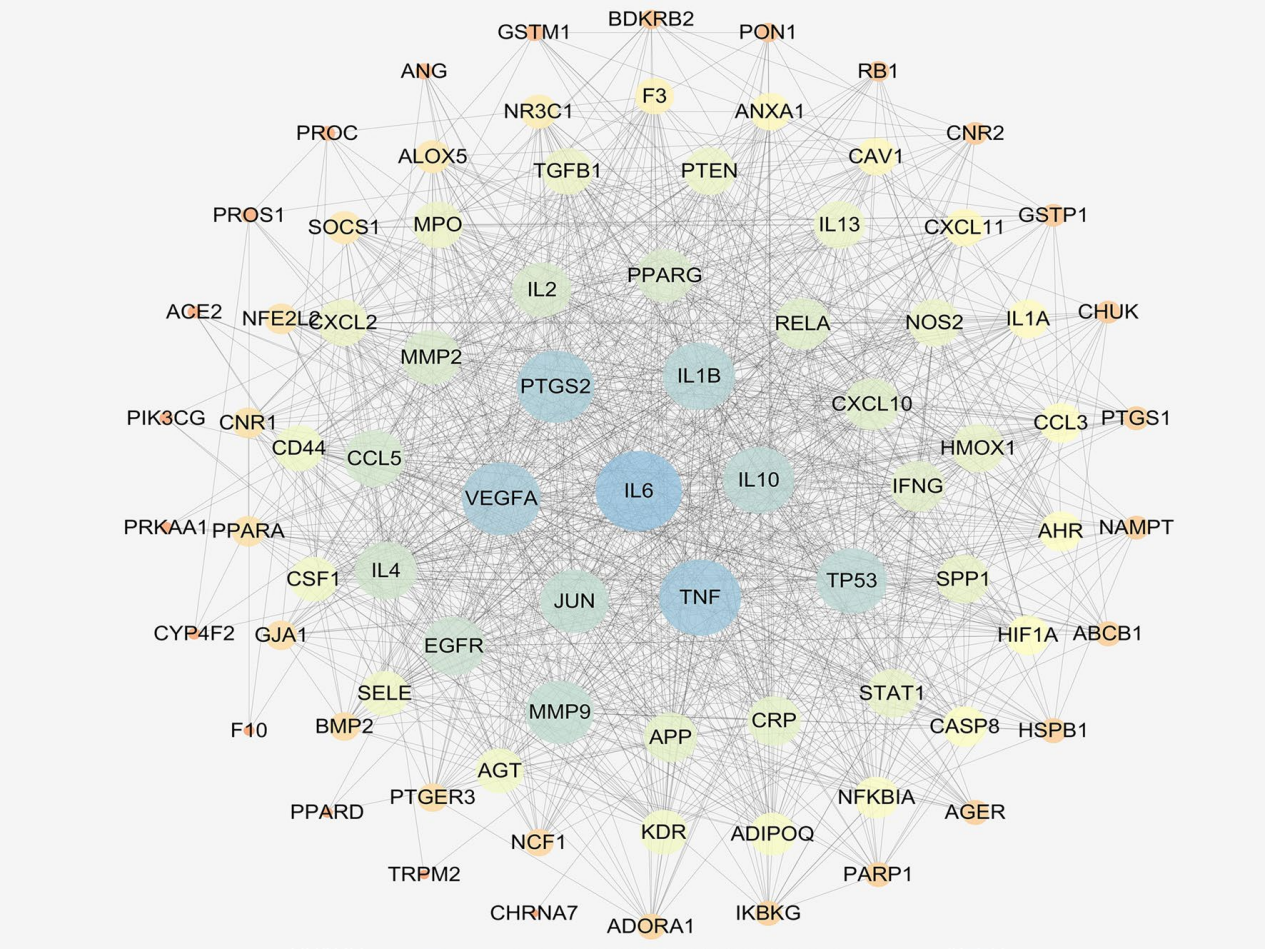
Protein–protein interaction (PPI) network analysis of Kushen for the treatment of inflammation using STRING database.

In order to clarify Kushen’s potential anti-inflammatory mechanisms, the obtained anti-inflammatory targets of the bioactive ingredients in Kushen were introduced into the STRING online database (PPI combined score > 0.7) to construct a PPI network, consisting of 81 nodes and 1088 interaction edges (Fig. 3). Based on the plug-in cytoHubba, the following hub genes were screened out in the PPI network according to the top five values of degree: IL-6, TNF-α, IL-1β, VEGFA, and PTGS2 (COX-2). Table 2 shows that these hub genes are mainly involved in enzymes, and signaling.

### Pathway enrichment analysis

**Figure 4:**
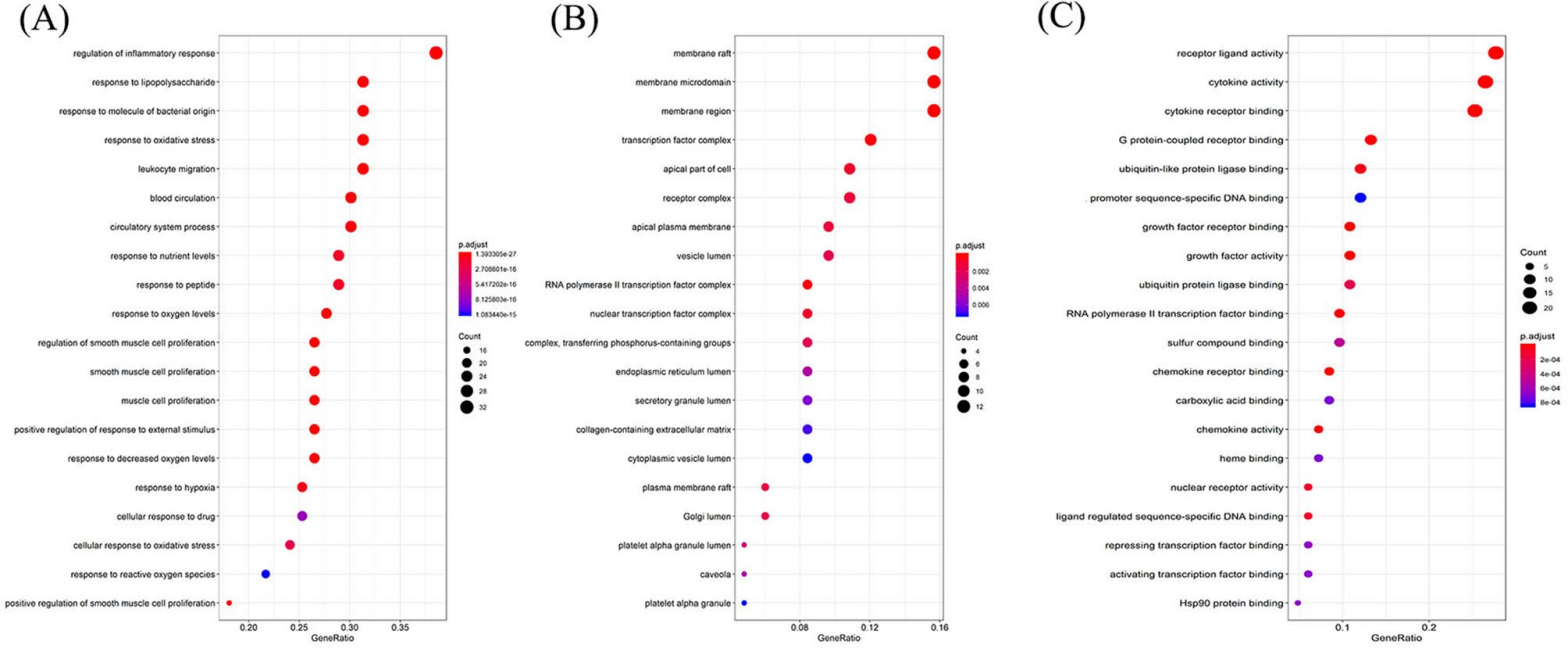
GO enrichment analysis of the putative targets. (**A**) The top 20 significant enriched terms in biological process (BP); (**B**) The top 20 significant enriched terms in cellular component (CC); (**C**) The top 20 significant enriched terms in molecular function (MF).

As shown in Fig. 4, GO enrichment analysis of the targets in the PPI network was performed using ClusterProfiler in R. The biological process (BP) results suggest that these targets respond to lipopolysaccharides, molecules of bacterial origin, oxidative stresses, nutrient levels, peptides, oxygen levels, decreased oxygen levels, reactive oxygen species, regulation of smooth muscles, and smooth muscle, muscle cell proliferation. Component cellular (CC) results included the vesicle, endoplasmic reticulum, secretory granule, Golgi, and platelet alpha granule lumen. For molecular function (MF), the targets mostly involved the binding of the cytokine receptor, G protein-coupled receptor, promoter sequence-specific DNA, growth factor receptor, and chemokine receptor. Furthermore, KEGG enrichment analysis has suggested that targets were mainly associated with the TNF signaling pathway, NF-kappa B signaling pathway, cytokine-cytokine receptor interaction, chemokine signaling pathway, and PI3K-Akt signaling pathway (Table 3).

**Table 3:** KEGG pathway enrichment analysis.

Description	Gene ratio	Bg Ratio	p value	p. adjust	q value
TNF signaling pathway	18/79	112/8033	1.33E−17	7.30E−16	3.01E−16
NF-kappa B signaling pathway	9/79	104/8033	6.99E−07	3.03E−06	1.25E−06
Toll-like receptor signaling pathway	16/79	104/8033	2.10E−15	7.13E−14	2.95E−14
Inflammatory bowel disease (IBD)	13/79	65/8033	3.15E−14	8.03E−13	3.32E−13
Cytokine-cytokine receptor interaction	18/79	294/8033	3.03E−10	3.25E−09	1.34E−09
Chemokine signaling pathway	14/79	189/8033	3.21E−09	2.52E−08	1.04E−08
T cell receptor signaling pathway	11/79	104/8033	4.35E−09	3.17E−08	1.31E−08
MAPK signaling pathway	15/79	294/8033	1.29E−07	7.31E−07	3.02E−07
PI3K-Akt signaling pathway	16/79	354/8033	2.46E−07	1.25E−06	5.17E−07
VEGF signaling pathway	5/79	59/8033	2.67E−04	6.9E−04	2.8E−04

### Effects of Kushen on cell viability and NO production

**Figure 5:**
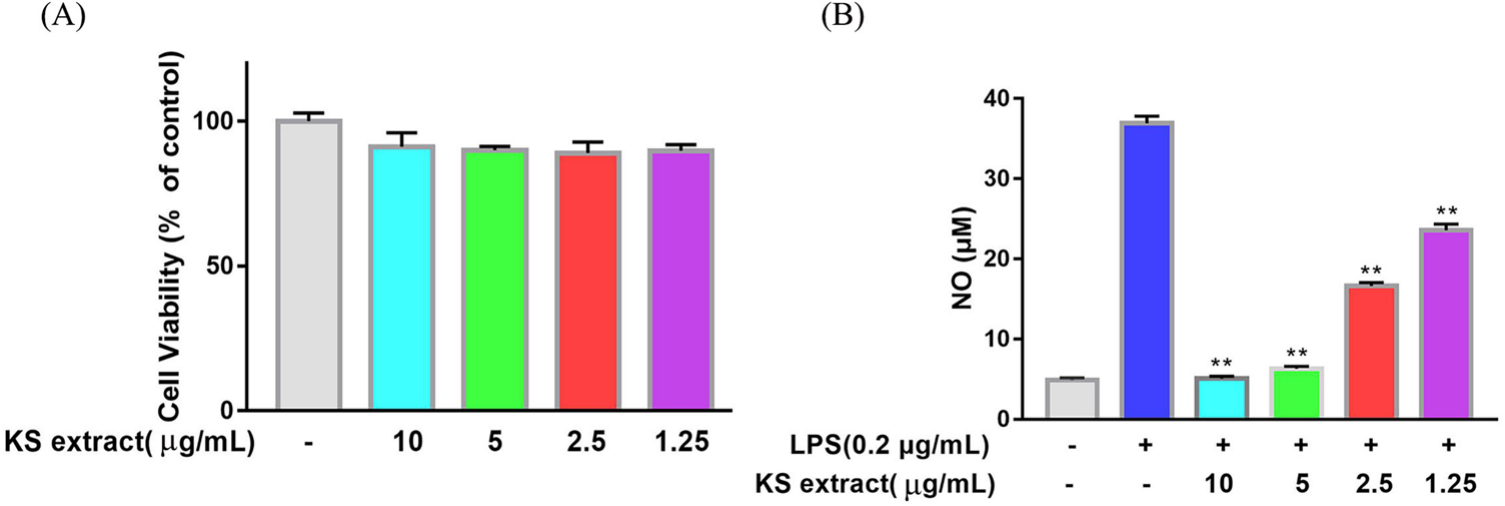
Effect of Kushen extract on RAW264.7 cells. (**A**) Cell viability of RAW264.7 cells after being treated by Kushen extract was detected by the CellTiter-LumiTM Plus, (**B**) NO production of LPS stimulated RAW264.7 cells after being treated with Kushen extract was detected by the Griess reaction. Data were presented as the mean ± SEM (n = 6), *p < 0.05 and **p < 0.01 versus LPS-treated group was considered statistically significant differences.

**Figure 6:**
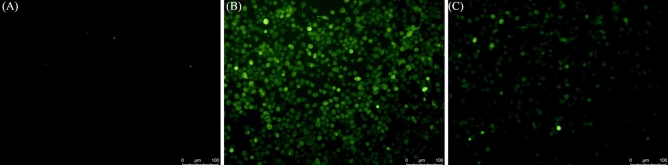
Effect of Kushen extract on intracellular NO production in RAW264.7 cells. The NO production of (**A**) Control group, (**B**) LPS-stimulated group, and (**C**) LPS-stimulated treated with Kushen extract group were determined by DAF-FM diacetate.

Previous studies have reported that the main components of Kushen, such as Matrine^15^, Oxymatrine^16^, Sophoridine^17^, Kushenol B^18^, own excellent anti-inflammatory effects. In this study, LPS-induced RAW264.7 cells inflammatory model was used cell to investigate the mechanism underlying Kushen’s anti-inflammatory effect on macrophagocytes. After an incubation period, the effects of Kushen extract on the viability of RAW264.7 cells were detected by CellTriter-Lumi ™ Plus. A viability assay showed that Kushen extract does not inhibit cell proliferation at any concentrations up to 10 μg/mL (Fig. 5A). In addition, the anti-inflammatory effects of Kushen extract on NO production in LPS-treated cells were detected by Griess reagent. As shown in Fig. 5B, the Kushen extract significantly inhibited NO production. Furthermore, laser microscopy showed that Kushen extract is a stronger inhibitor of intracellular NO production than LPS stimulation alone (Fig. 6).

### Suppression of the mRNA and protein expression of the hub genes by Kushen extract

**Figure 7:**
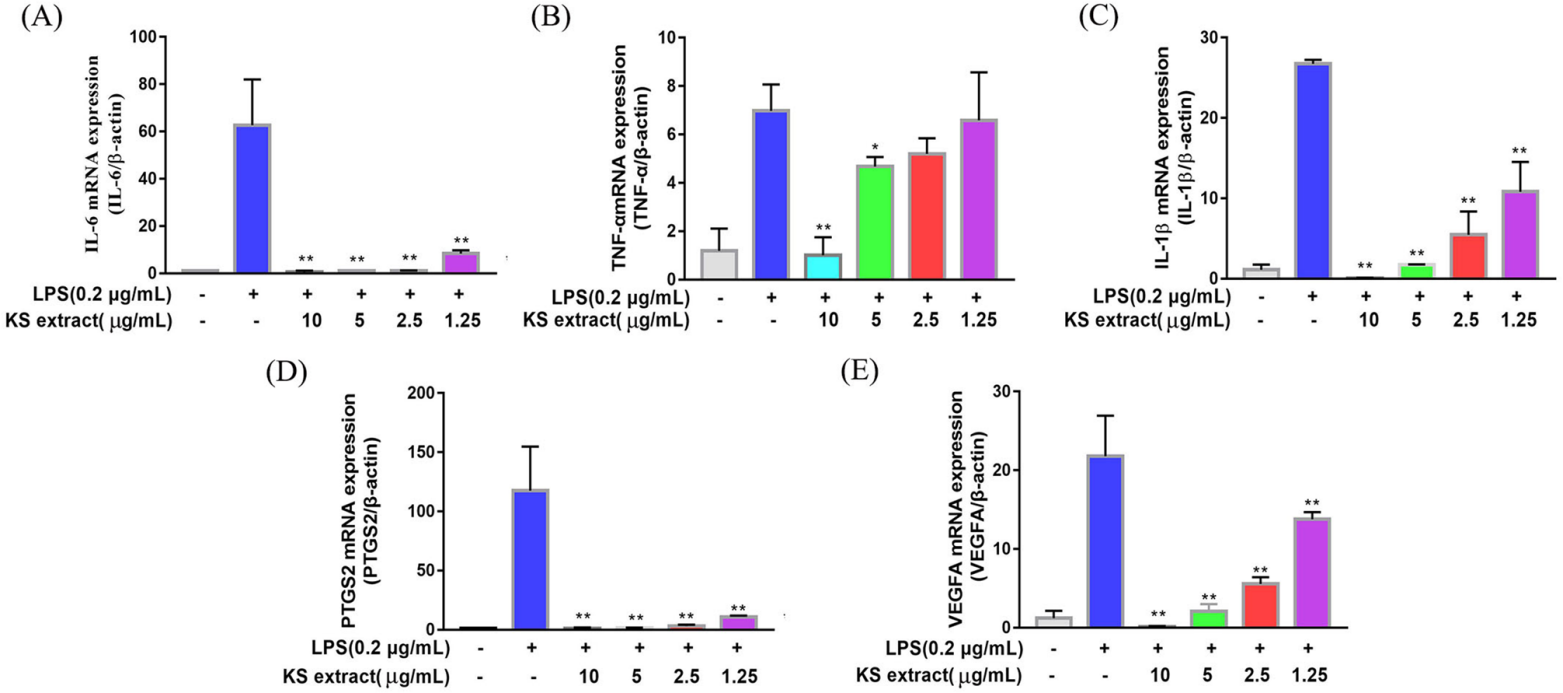
Effect of Kushen extract on key mRNA expression levels in RAW264.7 cells. The expression of (**A**) IL-6; (**B**) TNF-α; (**C**) IL-1β; (**D**) PTGS2 (COX-2); and (**E**) VEGFA mRNA levels were determined by RT-PCR. Data were presented as the mean ± SEM (n = 3), *p < 0.05 and **p < 0.01 versus LPS-treated group was considered statistically significant differences.

**Figure 8:**
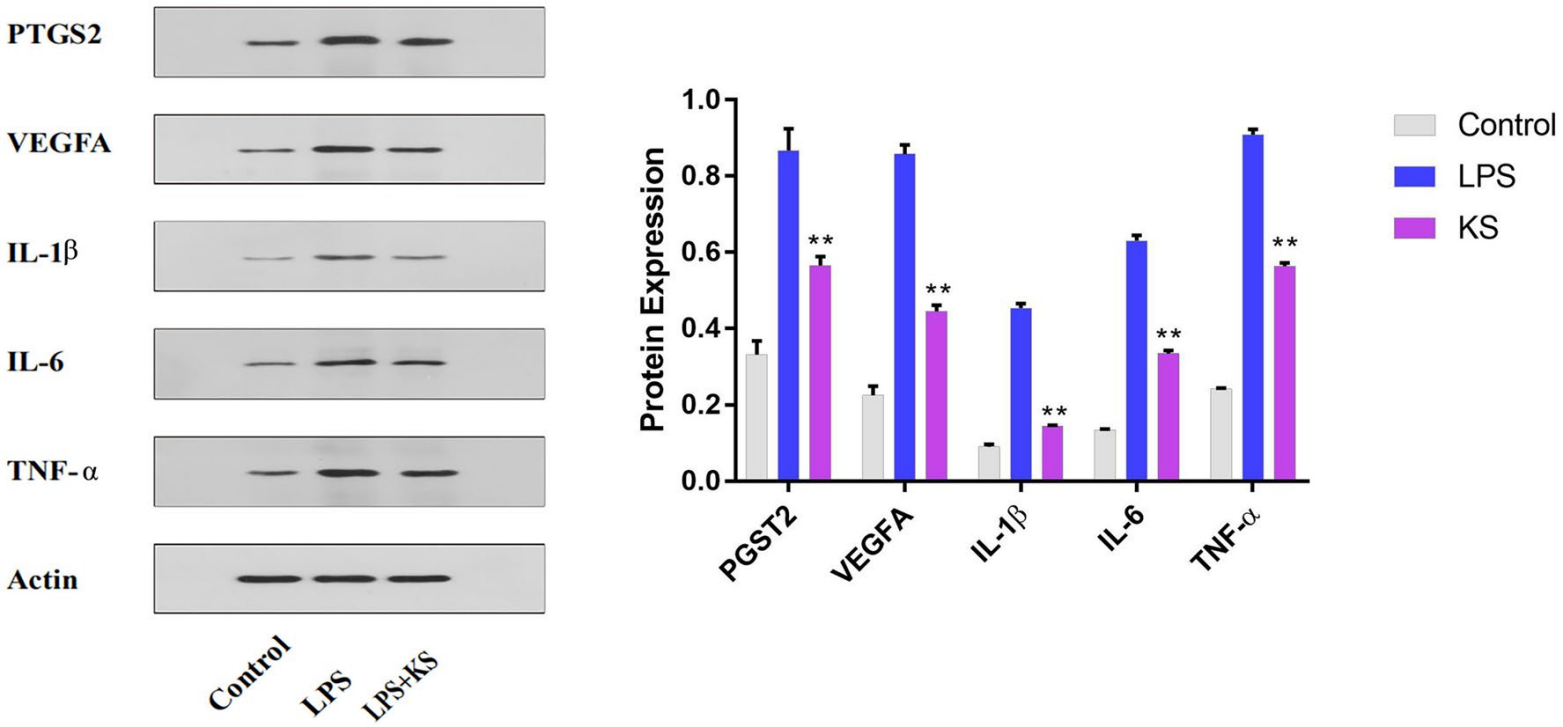
Effect of Kushen extract on key proteins expression levels in RAW264.7 cells. The expression of IL-6, TNF-α, IL-1β, PTGS2, and VEGFA protein levels were determined by western blot with specific antibodies and quantification. Data were presented as the mean ± SEM (n = 3), *p < 0.05 and **p < 0.01 versus LPS-treated group was considered statistically significant differences.

To investigate the effects of Kushen extract on the predicted hub genes by network pharmacology, the mRNA levels of *IL-6, IL-1β, VEGFA, TNF-α*, and *PTGS2 (COX-2)* were measured by quantitative real-time PCR, whereas the protein levels of IL-6, IL-1β, VEGFA, TNF-α, and PTGS2 (COX-2) were measured using western blot analysis. As shown in Fig. 7, the mRNA expression of *IL-6, IL-1β, VEGFA, TNF-α*, and *PTGS2 (COX-2)* was significantly increased after LPS stimulation (0.2 μg/mL). Moreover, the mRNA expression of these genes was significantly inhibited by all concentrations of Kushen extract in a concentration-dependent manner. In addition, the protein expression of *IL-6, IL-1β, VEGFA, TNF-α,* and *PTGS2 (COX-2)* in cells treated with Kushen extract was significantly inhibited compared to their expression in the LPS group alone (0.2 μg/mL) group (Fig. 8). Collectively, our study suggests that Kushen extract mainly treats inflammation by inhibiting these genes.

### Translocation of the NF-κB p65 subunit

**Figure 9:**
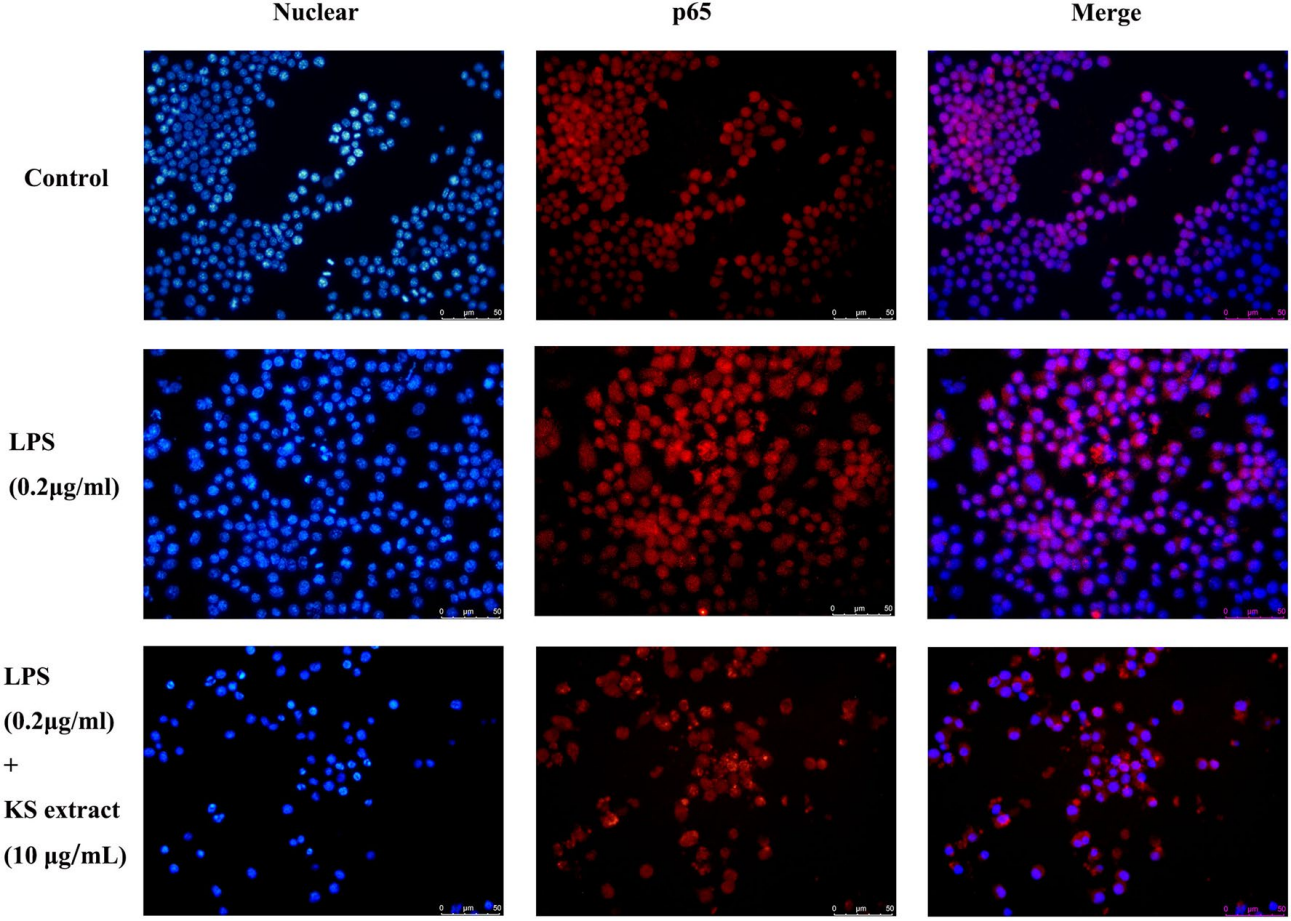
Effect of Kushen extract on the p65 subunit of NF-κB in RAW264.7 cells. The nuclear localization of the p65 subunit of NF-κB in (**A**) Control group, (**B**) LPS groups, and (**C**) LPS + Kushen extract were determined by immunofluorescence.

As shown in Table 4, the NF-κB signaling pathway is the key signaling pathway underlying the anti-inflammatory action of Kushen extract. The translocation of the NF-κB p65 subunit was determined by immunofluorescence. As shown in Fig. 9, after stimulation by LPS, p65 (red) was translocated from the cytoplasm to the nucleus; this clearly attenuated by Kushen extract (10 μg/mL), suggesting that the Kushen extract inhibited NF-κB activation.

**Table 4:** Primers used for the quantitative RT-PCR.

Gene	Primer	Sequence (5′–3′)
β-actin	Forward	TGTTACCAACTGGGACGACA
	Reverse	GGGGTGTTGAAGGTCTCAAA
COX-2	Forward	TGAGTACCGCAAACGCTTCTC
	Reverse	TGGACGAGGTTTTCCACCAG
TNF-α	Forward	TAGCCAGGAGGGAGAACAGA
	Reverse	TTTTCTGGAGGGAGATGTGG
IL-6	Forward	CTGGAGCCCACCAAGAACGA
	Reverse	GCCTCCGACTTGTGAAGTGGT
IL-1β	Forward	ATGCCACCTTTTGACAGTGATG
	Reverse	GTTGATGTGCTGCTGCGAGATT
VEGFA	Forward	TGAAGTGATCAAGTTCATGGACGT
	Reverse	TCACCGCCTTGGCTTGTC

## Discussion

Inflammation is a protective response against infection and injury^19^, which is closely linked to various chronic or malignant diseases, such as type II diabetes^20^, atherosclerosis^21^, and cancer^22^. Inflammation is a complex process involving multiple genes and signaling pathways^23^, and TCM is considered to have anti-inflammatory potential due to its effects via multiple-compounds, multiple-targets, and multiple pathways involved^24^. Kushen, well-known for its efficacy in clearing body heat, is a TCM mostly used to treat various syndrome that are caused by inflammation^25^ or infection^26^. Therefore, Kushen and its preparation, such as Kushen Lotion, Kushen Injection, are clinically used as adjuncts to treat inflammation-related diseases^27^. Through data mining and analysis, network pharmacology systematically interprets the overall relationship between drugs and targets, which perfectly fits TCM’s strategy of disease management through multiple ingredients, targets, and pathways^28^. Unlike previous studies, our study was the first to fully elucidate the anti-inflammatory mechanism of Kushen extract via network pharmacology methods and experimental validation, laying the foundation for further clinical research.

In this study, we retrieved 19 bioactive compounds of Kushen from the TCMSP, ETCM, and SymMap databases and five bioactive compounds with noteworthy pharmacological effects from literatures and found 465 predicted targets and used them to construct a “Component-Target” network. In the network, important compounds such as isosophocarpine (degree 218), quercetin (degree 207), dehydromiltirone (degree 166), and luteolin (degree 129) have a high degree value and are associated with many targets. Isosophocarpine, a tetracyclic quinolizidine alkaloid, shows anti-cancer effects on different types of cancer by attenuating inflammation^29^. Quercetin is a flavonoid with antioxidant, antiviral, and antibacterial effects, and is widely distributed in various plants and food. Lin et al.^30^ found that quercetin suppresses inflammation by countering the Azoxymethane/Dextran sodium sulfate (AOM/DSS)-induced carcinogenesis progression. In addition, Yue et al. have found that the dehydromiltirone is an anti-inflammatory compound that initiates p38 and the NF-κB signaling pathway in LPS-induced Kupffer cells^31^. Luteolin is a flavonoid commonly found in plants, such as celery, green pepper, honeysuckle, and chamomile. Previous studies have suggested that luteolin can reduce inflammation via inhibiting inflammation via activating the Nrf2/ARE, NF-κB, and MAPK signaling pathways^32^. These findings suggested that Kushen extract exerts its anti-inflammatory effects through multiple components and multiple targets.

A PPI network was constructed with 81 nodes and 1088 interaction edges to elucidate further the mechanisms of Kushen’s anti-inflammatory response. Then, the hub genes, namely IL-6, IL-1β, VEGFA, TNF-α, and PTGS2 (COX-2) were screened out based on the topological properties’ analysis. LPS-induced RAW264.7 cells constitute a typical inflammation model, and therefore the present study used them to investigate the anti-inflammatory effects of Kushen extract. A NO production assay showed that any concentration of Kushen extract inhibits the production of NO in a concentration-dependent manner, and intracellular NO production assay intuitively showed that Kushen extract shows maximum anti-inflammatory effect at 10 μg/mL. Moreover, RT-PCR and western blot further verified that the mRNA and protein levels of IL-6, IL-1β, VEGFA, TNF-α, and PTGS2 (COX-2) differ significantly between the LPS-induced and Kushen extract groups, suggesting that Kushen mainly exerts the anti-inflammatory effects via these predicted hub genes. IL-6, IL-1β, and TNF-α are crucial pro-inflammatory cytokines that coordinate a variety of inflammatory and immunomodulatory pathways that have broad effects on cells of the immune system^31,33^. VEGFA, a pleiotropic cytokine, has been considered as the indispensable part of angiogenesis, which mainly leads to cancer-related inflammation^34^. PTGS2 (COX-2), an enzyme induced by pro-inflammatory cytokines, releases prostaglandin E2 (PGE2) and promotes the synthesis of prostaglandins stimulating cancer cell proliferation, development, and metastasis; thus, it serves as a therapeutic target for anti-inflammatory drugs.

In, addition, the KEGG enrichment analysis of targets in the PPI network showed that Kushen’s anti-inflammatory effect is mainly enriched in the NF-κB signaling pathway and the PI3K-Akt signaling pathway. The PI3K-Akt/NF-κB signaling pathways play different roles in normal physiological responses and inflammatory processes^31,35^, including promoting cell proliferation, survival and differentiation. After activation of PI3K-Akt pathway, Akt enhances the phosphorylation and lowers the phosphorylation of the NF-κB inhibitor protein IκB kinase. PI3K/Akt/NF-κB signaling pathway server as the intersection of T and B cell inflammatory signaling pathway, resulting in increased expression of its maker proteins IKK-α, IκB-α, NF-κB p65, PI3K, p-AKT, p-NF-κB p65 in inflammatory model^36^. Among these, p65/RelA and p50 take important part in the NF-κB signaling pathway; p65 degrades when NF-κB signaling shuts down^37^. The immunofluorescence results also verified that Kushen extract (10 μg/mL) attenuates p65 from the cytoplasm to the nucleus in LPS-induced RAW264.7 cells, suggesting that the Kushen plays a crucial role in modulating inflammation via the NF-κB signaling pathway.

In conclusion, a network pharmacology approach was developed to elucidate the underlying molecular mechanism of anti-inflammatory effects of Kushen on inflammation. A total of 24 bioactive compounds, 465 Kushen-related targets, and 433 inflammation-related targets were obtained from open source databases. Furthermore, five hub genes were screened out based on a topological property analysis of the PPI network: IL-6, IL-1β, VEGFA, TNF-α, and PTGS2 (COX-2). Then, an experimental in vitro validation was performed to confirm the mRNA and protein expression of these hub genes and for enrichment analysis. Considering the complexity of the inflammatory process and TCM predicted targets, further research and clinical trials are necessary to confirm our findings.

## Materials and methods

### Reagents

Bacterial lipopolysaccharide (LPS) was obtained from Sigma. Dulbecco’s modified Eagle’s medium–high glucose (DMEM-HG) and heat inactivated fetal bovine serum (HI-FBS) were purchased from Biological Industries. Griess reagent system, DAPI, CellTiter-Lumi Plus Detection Kit, DAF-FM DA Detection Kit, and Cy3-labeled goat anti-rabbit IgG were obtained from Beyotime. RT-PCR kits were purchased from Takara Biotechnology Co., Ltd. Affinity provided the following antibodies: NF-κB p65, IL-6, IL-1β, TNF-α, VEGFA, PTGS2 (COX-2), and β-actin.

### Preparation of Kushen extract

Kushen herb was purchased from the Tongren Pharmaceutical Co., Ltd. (BEIJING, CHINA). Kushen (50 g) was soaked in 1 L of cold water for 30 min and then boiled for 30 min; this procedure was repeated three times. The combined extracts were concentrated to 1 g/mL (crude herbal dose) in a vacuum rotary evaporator. Then, the extract (1 mL) was filtered through microporous membrane before quantification.

### Network pharmacology analysis

#### Screening for active ingredients of Kushen

The bioactive ingredients of Kushen were screened from the following databases: Traditional Chinese Medicine Systems Pharmacology (TCMSP, https://tcmspw.com/tcmsp.php)^38^, the Encyclopedia of Traditional Chinese Medicine (ETCM, http://www.tcmip.cn/ETCM/index.php/Home/Index/All)^39^ and the SymMap (https://www.symmap.org/)^40^. According to the most common criteria of network pharmacology analysis^14^, the active ingredients with oral bioavailability (OB) ≥ 30% and drug-likeness (DL) ≥ 0.18 were selected for subsequent analysis.

#### Screening of potential targets for Kushen

Targets related to the candidate bioactive compounds of Kushen were collected from the ETCM, Search Tool for Interacting Chemicals (STITCH, http://stitch.embl.de/), SymMap, and Similarity ensemble approach (SEA, https://www.sogou.com/link?url=LeoKdSZoUyC9U6gWDurrLbchwv7HyEQP) databases^41^ with the *“Homo sapiens”* setting (Supplementary Excel 1).

#### Construction of a bioactive component-target network

To intuitively understand the mechanisms of Kushen extract treatment on anti-inflammation, a “Component-Target” network was constructed via Cytoscape 3.7.0 based on the bioactive components and predicted targets. In this network, the green rhombus node, blue round node, and edges indicate a bioactive component, a predicted target, and the interaction between the bioactive compounds and targets, respectively. The plug-in “Cytohubba” was applied to calculate the “degree” value of the node, which suggests the number of edges between the nodes in the network.

#### Screening for potential targets of inflammation

The keywords “inflammation,” “anti-inflammatory,” or “anti-inflammation” were used to search disease-related genes on the following databases: the Online Mendelian Inheritance in Man (OMIM, https://omim.org/), GeneCards (https://www.genecards.org/), and National Center for Biotechnology Information Gene Database (https://pubmed.ncbi.nlm.nih.gov/29140470/); the genes that overlapped among these databases were recorded (Supplementary Excel 2).

### Protein–protein interaction network construction and hub genes analysis

To further elucidate the potential mechanism underlying Kushen’s anti-inflammatory effect, a website was used to find overlapping inflammatory-related and predicted targets of Kushen. These overlapping targets were used to construct a protein–protein interaction (PPI) network on the Search Tool for the Retrieval of Interacting Genes/Proteins (STRING) database (https://string-db.org/) with the “*Homo sapiens*” setting. Cytoscape 3.7.0 was used to visualize PPI network, and the plug-in “Network Analysis” was performed to visualize the topological properties of each node in the network. To further elucidate the mechanism by which Kushen treats inflammation, the hub genes were screened out based on the topological properties of nodes in the PPI network. The plug-in “cytoHubba” was applied to calculate the value of degree in the PPI network, and the five genes with the highest values of degree were selected as anti-inflammatory hub genes for Kushen. Further, information on the target type (protein class) of the hub genes was taken from the DisGeNET database (https://www.disgenet.org).

### Gene ontology and KEGG pathway enrichment analyses

Gene ontology (GO) enrichment analysis is a bioinformatics tool for predicting gene function, while the Kyoto Encyclopedia of Genes and Genomes (KEGG, https://www.kegg.jp/) is a database for identifying the systematic functions and biological relevance of targets^42^. In order to analyze the biological pathways of genes in the PPI network, the “clusterProfiler” package (https://bioconductor.org/packages/release/bioc/html/clusterProfiler.html) in R (version: 3.6.3)^43^ was applied to analyze GO enrichment and KEGG pathway enrichment (adjusted to *P*. < 0.05).

### Cell culture

RAW264.7 cells were obtained from Cell Culture Center of the Chinese Academy of Medical Sciences. Cells were cultured in DMEM supplemented with 10% HI-FBS, 100 units/mL penicillin, and 100 μg/mL streptomycin at 37 °C in a fully humidified incubator containing 5% CO_2_.

### Cell viability assay

RAW264.7 cells were cultured in a 96-well plate at of 5 × 10^3^ cells/well and incubated overnight at 37 °C in an incubator containing 5% CO_2_. Subsequently, the cells were incubated with various concentrations of Kushen extract (10, 5, 2.5, and 1.25 μg/mL) for 24 h. Then, 100 μL of CellTiter-Lumi Plus detection reagent was added to each well, and the wells were vibrated for 5 min to fully lyse the cells completely. A luminometer (multifunctional microplate reader) was used to measure the luminescence (RLU) of each well. The viability of the Kushen extract was calculated as follows: (RLU_control_- RLU_treated_/RLU_control_) × 100%.

### Nitrite assay

RAW264.7 cells were cultured in 96-well plates at 1 × 10^4^ cells/well and treated as described above. NO production in the supernatant of the medium was measured by the Griess assay according to the manufacturer’s instructions and absorbance at 540 nm (OD_540_) was measured with a multifunctional microplate reader. In addition, DAF-FM was applied to qualitatively detect the concentration of NO. The cells were cultured and treated as described above. According to the instructions for the DAF-FM DA Kit, the images were generated with a Laser microscope (495 nm/515 nm).

### Total mRNA extraction and RT-PCR

Total RNA was isolated from cells treated with Kushen extract (10, 5, 2.5, and 1.25 μg/mL) and LPS (0.2 μg/mL) using Trizol reagent and reverse-transcribed into cDNA with PrimeScript II 1st Strand cDNA Synthesis Kit. The real-time PCR detection system and SYBR were applied to the PCR-amplified hub genes in accordance with the manufacture’s instruction manual. The primers for the hub genes are described in Table 4. β-Actin served as the internal control. The relative expression of mRNA was calculated as 2^−∆∆CT^.

### Protein extraction and western blot

Total protein was isolated from RAW264.7 cells treated with Kushen extract (10 μg/mL), and LPS (0.2 μg/mL) using RIPA lysis buffer: the protein concentration was quantified with a BCA protein assay kit as described previously^44^. Then, equal amounts of protein were separated by SDS-PAGE and transferred to PVDF membranes. After blocking in 0.01% Tween 20 containing 5% skimmed milk powder for 4 h, the membranes were incubated with a primary antibody (IL-6, IL-1β, PTGS2 (COX-2), TNF-α, VEGFA and β-actin) at 1:800 dilution overnight. Next, the membranes were incubated with anti-rabbit IgG secondary antibodies for 1 h. The blot bands were visualized and quantified using Gel Image system.

### Immunofluorescence assay

The total nuclear translocation of active p65 from the cytosol was assessed by immunofluorescence as described previously^44^. Briefly, RAW264.7 cells were stimulated with LPS (0.2 μg/mL) and treated with Kushen extract (10 μg/mL) for 6 h. Then, the cells were permeabilized with 0.1% Triton X-100 and incubated with anti-NF-κB p65 antibody (1:100 with 2% BSA) overnight at 4 °C. Next, the cells were incubated with Cy3-labeled goat anti-rabbit IgG, followed by DAPI mounting. Micrographs were captured under a fluorescence microscope.

### Statistical analysis

The data were analyzed with one-way analysis of variance (ANOVA) and *P* values < 0.05 were considered statistically significant. Statistical tests were performed using the GraphPad.

## Supplementary Information

Supplementary Information.

## Abbreviations

ANOVA: Analysis of variance
BP: Biological process
CC: Cell component
DL: Drug-likeness
DMEM: Dulbecco’s modified Eagle’s medium–high glucose
ETCM: Encyclopedia of Traditional Chinese Medicine
GO: Gene ontology
HI-FBS: Heat inactivated fetal bovine serum
IL-1β: Interleukin-1β
IRA: Inflammatory receptor agonist
KEGG: Kyoto Encyclopedia of Genes and Genomes
LPS: Lipopolysaccharide
MF: Molecular function
NSAIDs: Non-steroidal anti-inflammatory drugs
OB: Oral bioavailability
PGE2: Prostaglandin E2
PPI: Protein–protein interaction
SEA: Similarity ensemble approach
TNF-α: Tumor necrosis factor-α
TCM: Traditional Chinese Medicine
