# Supplementary material for: Molecular mechanism of the anti-inflammatory effects of Sophorae Flavescentis Aiton identified by network pharmacology
Source: Sci Rep. 2021 Jan 13;11:1005. doi: 10.1038/s41598-020-80297-y (PMC7806711; doi:10.1038/s41598-020-80297-y)

Molecular mechanism of the anti-inflammatory effects of Sophorae Flavescentis Aiton identified by network pharmacology

Suppelmentary Materials

Authors: Naiqiang Zhu, Jingyi Hou

Supplementary Material 1 Targets of bioactive compounds of Kushen.

**Target**
ADCY2
AKT1
APP
AR
BCL2L1
BIRC5
CASP3
CASP7
CCND1
CD40LG
CDK2
CDK4
CDKN1A
EGFR
ERBB2
GSTP1
HMOX1
ICAM1
IFNG
IL10
IL2
IL4
IL6
INSR
JUN
MAPK1
MAPK3
MCL1
MDM2
MET
MMP1
MMP2
MMP9
NFKBIA
NOS2
PCNA
PPARG
PRSS1
PTGS1
PTGS2
RB1
RELA

Target
TNF
TOP1
TOP2A
TP53
TYR
VEGFA
XDH
XIAP
CCNB1
CASP9
SLC2A4
PTGES
NUF2
ABCB1
ABCC1
ABCC2
ABCG2
ACTB
AHR
AKR1C1
ALB
ATP5A1
ATP5B
ATP5C1
CA1
CA12
CA14
CA2
CA3
CA4
CA5A
CA5B
CA6
CA7
CA9
CBR1
CDK6
CEBPB
COMT
CSNK2A1
CSNK2B
CYP19A1
Target

CYP1A1
CYP1A2
CYP1B1
CYP2C8
CYP2E1
EIF3F
ESR1
ESR2
ESRRA
ESRRB
GPER
HCK
HIBCH
HSP90AA1
HSPA2
IGHG1
JAK1
KIAA1310
MTTP
NCOA1
NCOA2
NQO2
NR1I2
PIK3CG
PIM1
PRKACA
PRKCA
PRKCB
PTK2B
RUVBL2
SF3B3
SHBG
SLC16A1
SLC16A7
SLC22A11
SLC22A6
SLCO2B1
SOAT1
SOAT2
SQLE
STK17B
SYK
Target
TTR

UBA1
UGT3A1
CALM3
CCNA2
GSK3B
KCNH2
KDR
RXRA
SCN5A
CHRNA3
CHRNA4
CHRNA6
CHRNA7
CHRNB2
CYP2D6
CYP3A4
HTR3A
TNF
CXCL8
ACACA
ACHE
ACPP
ADRB2
AKR1B1
ALOX5
BCL2
BMP2
CASP8
CAT
CAV1
CCL2
CDKN2A
CHEK2
CHUK
COL1A1
COL3A1
CRP
CTSD
CXCL10
DUOX2
EGF
EIF6
ERBB3
F3

FOS
GJA1
GSTM1
GSTM2
HIF1A
HSF1
HSPA5
HSPB1
IGF2
IGFBP3
IL1A
IL1B
IRF1
MAOB
MGAM
MMP3
MPO
MYC
NCF1
NOS3
NPEPPS
NQO1
NR1I3
ODC1
PARP1
PLAT
PLAU
PON1
POR
PPARA
PPARD
PTEN
PTGER3
RAF1
RASA1
RASSF1
RUNX2
SELE
SERPINE1
SOD1
STAT1
SULT1E1
TGFB1
THBD

VCAM1
ELK1
BAX
DIO1
E2F1
E2F2
CLDN4
RUNX1T1
HAS2
HK2
CXCL2
NFE2L2
NKX3-1
CXCL11
PCOLCE
PSMD3
SOD3
SPP1
AHSA1
DCAF5
HERC5
PRXC1A
CDC2
SEC14L3
PPP2CB
CNR1
SERPINE2
SREBF1
ABCB4
AFM
PPP2CA
SEC14L2
CHGA
SLC2A1
PTPN2
PKP3
ABCG1
DGKA
SEC14L6
JUP
CCL3
TTPA
PRKCG
REST

CNR2
FGF1
PRKAA1
NF2
SEC14L4
PKP2
SOCS1
PPP2R1A
RXRA
CYP3A5
CYP3A7
CYP2C19
CYP3A43
AAGAB
ADRA1A
ADRA2A
ADRB1
AKR1C3
APC
BAD
CCND2
CD44
CHRM1
CYCS
FCER2
FXYD2
G6PC
GRIA2
HSD3B1
HSD3B2
IER3IP1
IGF1R
IKBKG
IL13
INS
MAOA
MS4A2
MT-ND6
NR3C1
NR3C2
OPRM1
PGR
RXRB
SIRT1

SLC5A5
SLC6A2
SLC6A3
SLC6A4
TYRP1
DCT
ALPI
TRPM2
PSME3
CFLAR
EEF1E1
HPSE
ALG5
AWAT1
HTR4
GGCX
HTR1A
AGTR1
AVP
WLS
PCK1
CSF1
FNDC5
SLC38A3
RXFP4
MAPK9
MC4R
MTNR1B
MAS1
S1PR2
HEG1
CDH5
KCNK4
HTR3D
ANAPC2
BICD1
RET
SOD2
STATH
TMBIM6
SNCA
ADORA1
CCM2L
GABRA2

HTR1B
F10
AGTR2
VKORC1L1
PAX2
GPR27
ADCY6
IL34
ACIN1
CCL5
METRNL
LILRB1
CYP4F2
PPP1R1B
BMP6
EGR1
AGTRAP
TFPI
ARRB1
NEUROD2
WNT11
ACSL4
LRRK2
MC5R
HTR3E
NAPRT
NCK1
ALOX15
PROS1
GABRA3
VKORC1
BGLAP
F2
DAB2
SERPINB7
NAMPT
C1QTNF1
ANG
WT1
PLA2R1
PPP1R9B
PKD2
CRHBP
HTN1

SERPINF2
OXTR
CCS
AIF1
NMUR2
RPS6KA2
CDC20
MC3R
DRD5
MECOM
ADIPOQ
ANXA1
HTR3C
PROZ
F7
GABRA1
AGT
MGP
ACTN2
RGCC
MC2R
CD36
HNF1B
CLDN5
HTR2B
AQP2
CYP11A1
ACE2
SIPA1
SPRY1
GAS6
PPP1R15A
UTS2R
BDKRB2
CD47
SPX
THRB
HTR3B
RFK
AGER
CPLX2
UBIAD1
GLRA1
AVPR1A

F9
PROC
AVPR2
CBR4
NDRG2
HACD1
UTS2
SDC4
OXT
DCSTAMP
MC1R
PTGIS
CYP4F11
AMELX
HRH3
CTNNBIP1
PLA2G1B
ACVR2A
HCRT
ACE
ALOX15B
UGT3A2
REN
SPRY2
NCK2
FGA
AVPR1B
PRDM16
DRD2
PAWR

Supplementary Materials 2 Targets of inflammation

Target	Source
A4GNT	OMIM,Genecards, Gene
ABCB1	OMIM,Genecards, Gene
ACE2	OMIM,Genecards, Gene
ACKR1	OMIM,Genecards, Gene
ACKR2	OMIM,Genecards, Gene
ACKR3	OMIM,Genecards, Gene
ACP5	OMIM,Genecards, Gene
ACVR1	OMIM,Genecards, Gene
ADA	OMIM,Genecards, Gene
ADAMTS12	OMIM,Genecards, Gene
ADAR	OMIM,Genecards, Gene
ADCYAP1	OMIM,Genecards, Gene
ADGRE2	OMIM,Genecards, Gene
ADIPOQ	OMIM,Genecards, Gene
ADIPOR1	OMIM,Genecards, Gene
ADIPOR2	OMIM,Genecards, Gene
ADORA1	OMIM,Genecards, Gene
ADORA2A	OMIM,Genecards, Gene
AGER	OMIM,Genecards, Gene
AGT	OMIM,Genecards, Gene
AHR	OMIM,Genecards, Gene
AIM2	OMIM,Genecards, Gene
AIMP1	OMIM,Genecards, Gene
AKT1	OMIM,Genecards, Gene
ALOX5	OMIM,Genecards, Gene
ALPK1	OMIM,Genecards, Gene
ANG	OMIM,Genecards, Gene
ANGPT1	OMIM,Genecards, Gene
ANGPT2	OMIM,Genecards, Gene
ANXA1	OMIM,Genecards, Gene
APP	OMIM,Genecards, Gene
ARG1	OMIM,Genecards, Gene
ATG16L1	OMIM,Genecards, Gene
ATG5	OMIM,Genecards, Gene
AXL	OMIM,Genecards, Gene
BACH2	OMIM,Genecards, Gene
BCL6	OMIM,Genecards, Gene
BDKRB1	OMIM,Genecards, Gene
BDKRB2	OMIM,Genecards, Gene
BDNF	OMIM,Genecards, Gene
BMP2	OMIM,Genecards, Gene
BTLA	OMIM,Genecards, Gene
**Target**	**Source**

C1QBP OMIM,Genecards, Gene
C3 OMIM,Genecards, Gene
C5 OMIM,Genecards, Gene
C5AR1 OMIM,Genecards, Gene
C5AR2 OMIM,Genecards, Gene
CACNA1C OMIM,Genecards, Gene
CAMP OMIM,Genecards, Gene
CAPN5 OMIM,Genecards, Gene
CARD8 OMIM,Genecards, Gene
CASP1 OMIM,Genecards, Gene
CASP8 OMIM,Genecards, Gene
CAV1 OMIM,Genecards, Gene
CCL11 OMIM,Genecards, Gene
CCL13 OMIM,Genecards, Gene
CCL18 OMIM,Genecards, Gene
CCL19 OMIM,Genecards, Gene
CCL2 OMIM,Genecards, Gene
CCL20 OMIM,Genecards, Gene
CCL21 OMIM,Genecards, Gene
CCL22 OMIM,Genecards, Gene
CCL27 OMIM,Genecards, Gene
CCL3 OMIM,Genecards, Gene
CCL5 OMIM,Genecards, Gene
CCL7 OMIM,Genecards, Gene
CCR1 OMIM,Genecards, Gene
CCR10 OMIM,Genecards, Gene
CCR2 OMIM,Genecards, Gene
CCR3 OMIM,Genecards, Gene
CCR4 OMIM,Genecards, Gene
CCR5 OMIM,Genecards, Gene
CCR6 OMIM,Genecards, Gene
CCR8 OMIM,Genecards, Gene
CCR9 OMIM,Genecards, Gene
CD14 OMIM,Genecards, Gene
CD163 OMIM,Genecards, Gene
CD1D OMIM,Genecards, Gene
CD300LF OMIM,Genecards, Gene
CD4 OMIM,Genecards, Gene
CD40 OMIM,Genecards, Gene
CD44 OMIM,Genecards, Gene
CD80 OMIM,Genecards, Gene
CD99 OMIM,Genecards, Gene
CDH11 OMIM,Genecards, Gene
CDH26 OMIM,Genecards, Gene

CDK5	OMIM,Genecards, Gene
CERK	OMIM,Genecards, Gene
CFB	OMIM,Genecards, Gene
CFH	OMIM,Genecards, Gene
CFTR	OMIM,Genecards, Gene
CHI3L1	OMIM,Genecards, Gene
CHRNA7	OMIM,Genecards, Gene
CHUK	OMIM,Genecards, Gene
CLEC10A	OMIM,Genecards, Gene
CLEC4D	OMIM,Genecards, Gene
CLEC4E	OMIM,Genecards, Gene
CLEC5A	OMIM,Genecards, Gene
CLU	OMIM,Genecards, Gene
CMA1	OMIM,Genecards, Gene
CMKLR1	OMIM,Genecards, Gene
CNR1	OMIM,Genecards, Gene
CNR2	OMIM,Genecards, Gene
CPB2	OMIM,Genecards, Gene
CR1	OMIM,Genecards, Gene
CRH	OMIM,Genecards, Gene
CRP	OMIM,Genecards, Gene
CRYAB	OMIM,Genecards, Gene
CSF1	OMIM,Genecards, Gene
CSF2	OMIM,Genecards, Gene
CST9	OMIM,Genecards, Gene
CTLA4	OMIM,Genecards, Gene
CTNND1	OMIM,Genecards, Gene
CTSG	OMIM,Genecards, Gene
CTSK	OMIM,Genecards, Gene
CX3CL1	OMIM,Genecards, Gene
CX3CR1	OMIM,Genecards, Gene
CXCL10	OMIM,Genecards, Gene
CXCL11	OMIM,Genecards, Gene
CXCL12	OMIM,Genecards, Gene
CXCL13	OMIM,Genecards, Gene
CXCL14	OMIM,Genecards, Gene
CXCL2	OMIM,Genecards, Gene
CXCR1	OMIM,Genecards, Gene
CXCR2	OMIM,Genecards, Gene
CXCR3	OMIM,Genecards, Gene
CXCR4	OMIM,Genecards, Gene
CXCR6	OMIM,Genecards, Gene
CYBB	OMIM,Genecards, Gene

CYP4F2	OMIM,Genecards, Gene
CYP4F3	OMIM,Genecards, Gene
CYSLTR1	OMIM,Genecards, Gene
CYSLTR2	OMIM,Genecards, Gene
CYTH2	OMIM,Genecards, Gene
DDIT3	OMIM,Genecards, Gene
DDIT4	OMIM,Genecards, Gene
DEFA5	OMIM,Genecards, Gene
DEFB1	OMIM,Genecards, Gene
DEFB4A	OMIM,Genecards, Gene
DPP4	OMIM,Genecards, Gene
DUSP1	OMIM,Genecards, Gene
EBI3	OMIM,Genecards, Gene
EDIL3	OMIM,Genecards, Gene
EDN1	OMIM,Genecards, Gene
EGFR	OMIM,Genecards, Gene
EIF2AK2	OMIM,Genecards, Gene
ELANE	OMIM,Genecards, Gene
ENTPD1	OMIM,Genecards, Gene
EOMES	OMIM,Genecards, Gene
EPO	OMIM,Genecards, Gene
EREG	OMIM,Genecards, Gene
ETS1	OMIM,Genecards, Gene
F10	OMIM,Genecards, Gene
F12	OMIM,Genecards, Gene
F2R	OMIM,Genecards, Gene
F2RL1	OMIM,Genecards, Gene
F3	OMIM,Genecards, Gene
FABP4	OMIM,Genecards, Gene
FADD	OMIM,Genecards, Gene
FAS	OMIM,Genecards, Gene
FASLG	OMIM,Genecards, Gene
FASN	OMIM,Genecards, Gene
FCER1A	OMIM,Genecards, Gene
FCGR2B	OMIM,Genecards, Gene
FCGR3A	OMIM,Genecards, Gene
FERMT1	OMIM,Genecards, Gene
FETUB	OMIM,Genecards, Gene
FFAR2	OMIM,Genecards, Gene
FFAR4	OMIM,Genecards, Gene
FLG	OMIM,Genecards, Gene
FLT1	OMIM,Genecards, Gene
FOXP3	OMIM,Genecards, Gene
FPR1	OMIM,Genecards, Gene

FPR2	OMIM,Genecards, Gene
GATA4	OMIM,Genecards, Gene
GATA6	OMIM,Genecards, Gene
GCG	OMIM,Genecards, Gene
GCH1	OMIM,Genecards, Gene
GDF15	OMIM,Genecards, Gene
GHRL	OMIM,Genecards, Gene
GHSR	OMIM,Genecards, Gene
GJA1	OMIM,Genecards, Gene
GRN	OMIM,Genecards, Gene
GSN	OMIM,Genecards, Gene
GSTM1	OMIM,Genecards, Gene
GSTP1	OMIM,Genecards, Gene
GUCA2B	OMIM,Genecards, Gene
HAMP	OMIM,Genecards, Gene
HAVCR1	OMIM,Genecards, Gene
HCAR2	OMIM,Genecards, Gene
HIF1A	OMIM,Genecards, Gene
HMGB1	OMIM,Genecards, Gene
HMOX1	OMIM,Genecards, Gene
HPGD	OMIM,Genecards, Gene
HRH1	OMIM,Genecards, Gene
HRH2	OMIM,Genecards, Gene
HS6ST3	OMIM,Genecards, Gene
HSPA1A	OMIM,Genecards, Gene
HSPB1	OMIM,Genecards, Gene
ICAM3	OMIM,Genecards, Gene
ICOS	OMIM,Genecards, Gene
ICOSLG	OMIM,Genecards, Gene
IDO1	OMIM,Genecards, Gene
IFNG	OMIM,Genecards, Gene
IGF1	OMIM,Genecards, Gene
IKBKB	OMIM,Genecards, Gene
IKBKE	OMIM,Genecards, Gene
IKBKG	OMIM,Genecards, Gene
IL10	OMIM,Genecards, Gene
IL11	OMIM,Genecards, Gene
IL13	OMIM,Genecards, Gene
IL15	OMIM,Genecards, Gene
IL16	OMIM,Genecards, Gene
IL18	OMIM,Genecards, Gene
IL1A	OMIM,Genecards, Gene
IL1B	OMIM,Genecards, Gene
IL2	OMIM,Genecards, Gene

IL21	OMIM,Genecards, Gene
IL22	OMIM,Genecards, Gene
IL22RA1	OMIM,Genecards, Gene
IL23A	OMIM,Genecards, Gene
IL24	OMIM,Genecards, Gene
IL27	OMIM,Genecards, Gene
IL2RA	OMIM,Genecards, Gene
IL2RB	OMIM,Genecards, Gene
IL31	OMIM,Genecards, Gene
IL32	OMIM,Genecards, Gene
IL33	OMIM,Genecards, Gene
IL36A	OMIM,Genecards, Gene
IL36B	OMIM,Genecards, Gene
IL36G	OMIM,Genecards, Gene
IL36RN	OMIM,Genecards, Gene
IL4	OMIM,Genecards, Gene
IL5	OMIM,Genecards, Gene
IL6	OMIM,Genecards, Gene
IL9	OMIM,Genecards, Gene
IRAK3	OMIM,Genecards, Gene
IRF5	OMIM,Genecards, Gene
ISG15	OMIM,Genecards, Gene
ITGA1	OMIM,Genecards, Gene
ITGAM	OMIM,Genecards, Gene
ITGB1	OMIM,Genecards, Gene
ITGB2	OMIM,Genecards, Gene
JUN	OMIM,Genecards, Gene
JUND	OMIM,Genecards, Gene
KDR	OMIM,Genecards, Gene
KLRB1	OMIM,Genecards, Gene
KLRK1	OMIM,Genecards, Gene
KMT2A	OMIM,Genecards, Gene
LBP	OMIM,Genecards, Gene
LCN2	OMIM,Genecards, Gene
LEP	OMIM,Genecards, Gene
LGALS2	OMIM,Genecards, Gene
LGALS3	OMIM,Genecards, Gene
LGR5	OMIM,Genecards, Gene
LST1	OMIM,Genecards, Gene
LTB4R	OMIM,Genecards, Gene
LTB4R2	OMIM,Genecards, Gene
LTBR	OMIM,Genecards, Gene
MAP3K14	OMIM,Genecards, Gene
MAP3K3	OMIM,Genecards, Gene

MAP3K8	OMIM,Genecards, Gene
MAP4K4	OMIM,Genecards, Gene
MAPK14	OMIM,Genecards, Gene
MAPK8	OMIM,Genecards, Gene
MAVS	OMIM,Genecards, Gene
MAZ	OMIM,Genecards, Gene
MEFV	OMIM,Genecards, Gene
MEP1B	OMIM,Genecards, Gene
MERTK	OMIM,Genecards, Gene
MIF	OMIM,Genecards, Gene
MITF	OMIM,Genecards, Gene
MMP12	OMIM,Genecards, Gene
MMP2	OMIM,Genecards, Gene
MMP9	OMIM,Genecards, Gene
MPO	OMIM,Genecards, Gene
MST1R	OMIM,Genecards, Gene
MUC5AC	OMIM,Genecards, Gene
MUC5B	OMIM,Genecards, Gene
MVK	OMIM,Genecards, Gene
MYD88	OMIM,Genecards, Gene
NAMPT	OMIM,Genecards, Gene
NCF1	OMIM,Genecards, Gene
NCR3	OMIM,Genecards, Gene
NFE2L1	OMIM,Genecards, Gene
NFE2L2	OMIM,Genecards, Gene
NFKB1	OMIM,Genecards, Gene
NFKBIA	OMIM,Genecards, Gene
NFKBIZ	OMIM,Genecards, Gene
NLRC4	OMIM,Genecards, Gene
NLRP1	OMIM,Genecards, Gene
NLRP2	OMIM,Genecards, Gene
NLRP3	OMIM,Genecards, Gene
NLRX1	OMIM,Genecards, Gene
NOD1	OMIM,Genecards, Gene
NOD2	OMIM,Genecards, Gene
NOTCH1	OMIM,Genecards, Gene
NOX1	OMIM,Genecards, Gene
NR1H2	OMIM,Genecards, Gene
NR1H3	OMIM,Genecards, Gene
NR3C1	OMIM,Genecards, Gene
NR4A2	OMIM,Genecards, Gene
NR5A2	OMIM,Genecards, Gene
OCLN	OMIM,Genecards, Gene
OPTN	OMIM,Genecards, Gene

OTULIN	OMIM,Genecards, Gene
P2RX7	OMIM,Genecards, Gene
P2RY6	OMIM,Genecards, Gene
PARP1	OMIM,Genecards, Gene
PDCD1	OMIM,Genecards, Gene
PDPN	OMIM,Genecards, Gene
PECAM1	OMIM,Genecards, Gene
PF4	OMIM,Genecards, Gene
PGF	OMIM,Genecards, Gene
PIK3CG	OMIM,Genecards, Gene
PIN1	OMIM,Genecards, Gene
PLA2G3	OMIM,Genecards, Gene
PLA2G4A	OMIM,Genecards, Gene
PLA2G7	OMIM,Genecards, Gene
PLG	OMIM,Genecards, Gene
PON1	OMIM,Genecards, Gene
PON3	OMIM,Genecards, Gene
PPARA	OMIM,Genecards, Gene
PPARD	OMIM,Genecards, Gene
PPARG	OMIM,Genecards, Gene
PRKAA1	OMIM,Genecards, Gene
PRKCD	OMIM,Genecards, Gene
PROC	OMIM,Genecards, Gene
PROK2	OMIM,Genecards, Gene
PROS1	OMIM,Genecards, Gene
PRTN3	OMIM,Genecards, Gene
PTEN	OMIM,Genecards, Gene
PTGDR2	OMIM,Genecards, Gene
PTGER3	OMIM,Genecards, Gene
PTGER4	OMIM,Genecards, Gene
PTGIR	OMIM,Genecards, Gene
PTGS1	OMIM,Genecards, Gene
PTGS2	OMIM,Genecards, Gene
PTPN6	OMIM,Genecards, Gene
PYCARD	OMIM,Genecards, Gene
RAC1	OMIM,Genecards, Gene
RALBP1	OMIM,Genecards, Gene
RB1	OMIM,Genecards, Gene
RC3H1	OMIM,Genecards, Gene
REG1A	OMIM,Genecards, Gene
REG4	OMIM,Genecards, Gene
RELA	OMIM,Genecards, Gene
RETN	OMIM,Genecards, Gene
RIPK1	OMIM,Genecards, Gene

RIPK3	OMIM,Genecards, Gene
RIPK4	OMIM,Genecards, Gene
ROCK1	OMIM,Genecards, Gene
RORC	OMIM,Genecards, Gene
RUNX3	OMIM,Genecards, Gene
S100A12	OMIM,Genecards, Gene
S100A8	OMIM,Genecards, Gene
S100A9	OMIM,Genecards, Gene
S1PR1	OMIM,Genecards, Gene
SAMHD1	OMIM,Genecards, Gene
SCGB1A1	OMIM,Genecards, Gene
SCGB3A2	OMIM,Genecards, Gene
SELE	OMIM,Genecards, Gene
SELL	OMIM,Genecards, Gene
SELP	OMIM,Genecards, Gene
SELPLG	OMIM,Genecards, Gene
SEMA7A	OMIM,Genecards, Gene
SERPINA1	OMIM,Genecards, Gene
SERPINB2	OMIM,Genecards, Gene
SERPINB3	OMIM,Genecards, Gene
SERPINB4	OMIM,Genecards, Gene
SFTPA1	OMIM,Genecards, Gene
SFTPD	OMIM,Genecards, Gene
SGK1	OMIM,Genecards, Gene
SIGIRR	OMIM,Genecards, Gene
SLC11A2	OMIM,Genecards, Gene
SLC22A5	OMIM,Genecards, Gene
SMAD4	OMIM,Genecards, Gene
SOCS1	OMIM,Genecards, Gene
SOCS3	OMIM,Genecards, Gene
SPINK1	OMIM,Genecards, Gene
SPP1	OMIM,Genecards, Gene
SRC	OMIM,Genecards, Gene
SST	OMIM,Genecards, Gene
ST14	OMIM,Genecards, Gene
STAT1	OMIM,Genecards, Gene
STAT3	OMIM,Genecards, Gene
STAT4	OMIM,Genecards, Gene
STAT6	OMIM,Genecards, Gene
STK11	OMIM,Genecards, Gene
TAC1	OMIM,Genecards, Gene
TAP2	OMIM,Genecards, Gene
TBX21	OMIM,Genecards, Gene
TET2	OMIM,Genecards, Gene

TFRC	OMIM,Genecards, Gene
TGFB1	OMIM,Genecards, Gene
TGFBR2	OMIM,Genecards, Gene
TICAM1	OMIM,Genecards, Gene
TIE1	OMIM,Genecards, Gene
TIMP3	OMIM,Genecards, Gene
TIRAP	OMIM,Genecards, Gene
TLR1	OMIM,Genecards, Gene
TLR2	OMIM,Genecards, Gene
TLR3	OMIM,Genecards, Gene
TLR4	OMIM,Genecards, Gene
TLR5	OMIM,Genecards, Gene
TLR7	OMIM,Genecards, Gene
TLR9	OMIM,Genecards, Gene
TNC	OMIM,Genecards, Gene
TNF	OMIM,Genecards, Gene
TP53	OMIM,Genecards, Gene
TP63	OMIM,Genecards, Gene
TRAF1	OMIM,Genecards, Gene
TRAF3IP2	OMIM,Genecards, Gene
TRAF6	OMIM,Genecards, Gene
TREM1	OMIM,Genecards, Gene
TREM2	OMIM,Genecards, Gene
TRIM27	OMIM,Genecards, Gene
TRPA1	OMIM,Genecards, Gene
TRPM2	OMIM,Genecards, Gene
TRPM8	OMIM,Genecards, Gene
TRPV1	OMIM,Genecards, Gene
TSC22D3	OMIM,Genecards, Gene
TSLP	OMIM,Genecards, Gene
TYRO3	OMIM,Genecards, Gene
TYROBP	OMIM,Genecards, Gene
VDR	OMIM,Genecards, Gene
VEGFA	OMIM,Genecards, Gene
VEGFB	OMIM,Genecards, Gene
VLDLR	OMIM,Genecards, Gene
WWTR1	OMIM,Genecards, Gene
XBP1	OMIM,Genecards, Gene
YAP1	OMIM,Genecards, Gene
ZFP36	OMIM,Genecards, Gene

Supplementary Material 3. The fuller-length originals of Western blot

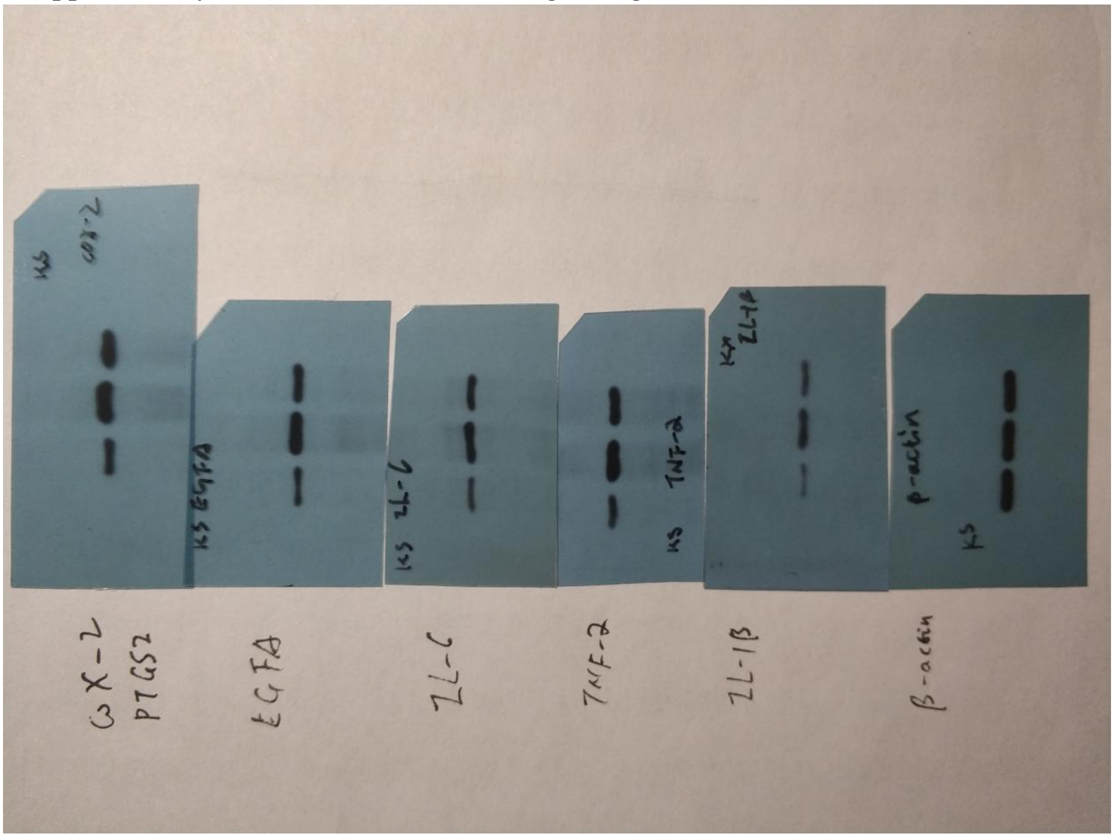

Supplement: Supplementary file 1 — Supplementary Information. [file 41598_2020_80297_MOESM1_ESM.pdf]
